# The Effect of BPA-Treated Water on the Small Intestine via an In Vivo Study

**DOI:** 10.3390/toxics10060296

**Published:** 2022-05-30

**Authors:** Roziana Kamaludin, Zatilfarihiah Rasdi, Mohd Hafiz Dzarfan Othman, Siti Hamimah Sheikh Abdul Kadir, Mohd Yusri Idorus, Jesmine Khan, Wan Nor I’zzah Wan Mohamad Zain, Ahmad Fauzi Ismail, Mukhlis A. Rahman, Juhana Jaafar

**Affiliations:** 1 Advanced Membrane Technology Research Centre (AMTEC), Universiti Teknologi Malaysia, Skudai 81310, Malaysia; roziana.kamaludin7@gmail.com (R.K.); afauzi@utm.my (A.F.I.); mukhlis@petroleum.utm.my (M.A.R.); juhana@petroleum.utm.my (J.J.); 2 Centre of Preclinical Sciences Studies, Faculty of Dentistry, Universiti Teknologi MARA (UiTM), Jalan Hospital, Sungai Buloh 47000, Malaysia; zatilfarihiah@uitm.edu.my; 3 Sungai Buloh Campus, Institute of Pathology, Laboratory and Forensic Medicine (I-PPerForM), Universiti Teknologi MARA (UiTM), Jalan Hospital, Sungai Buloh 47000, Malaysia; 4 Biochemistry and Molecular Medicine Department, Faculty of Medicine, Sungai Buloh Campus, Universiti Teknologi MARA (UiTM), Jalan Hospital, Sungai Buloh 47000, Malaysia; jesminek@salam.uitm.edu.my (J.K.); wnizzah@salam.uitm.edu.my (W.N.I.W.M.Z.); 5 Faculty of Medicine, Sungai Buloh Campus, Institute of Medical Molecular Biotechnology, Universiti Teknologi MARA (UiTM), Jalan Hospital, Sungai Buloh 47000, Malaysia; myusri@uitm.edu.my

**Keywords:** bisphenol A, photocatalytic, gastrointestinal barrier, tight junction protein, claudin, small intestine

## Abstract

Since the major route of BPA exposure is via the oral route, BPA may have effects on the gastrointestinal tract, especially on the intestinal barrier, where most digestion and absorption processes occur. In this study, the effects of BPA-treated water on the small intestine (SI) and SI tight junction proteins (TJPs) of both pregnant Sprague–Dawley rats and their fetuses were investigated. Previously, hybrid photocatalytic filtration treatment by a visible light driven N-doped TiO_2_ membrane has successfully removed up to 81.6% of BPA in water. The effect of BPA-untreated (5.00 ± ppm) and BPA-treated water (0.9 ± ppm) after 21 days of exposure on the jejunum and ileum, as well as the expressions of claudin proteins, were investigated by Western blotting (WB) and hematoxylin and eosin (H&E) in order to investigate the potential of the photocatalytic membrane in removing the detrimental effect of BPA. The results suggest that BPA exposure altered the morphology of villi, and affected the expression level of claudin-2, -3, and -4 proteins in the jejunum and ileum of both pregnant rats and their fetuses. Interestingly, villi and claudins expressions were undisrupted in a treated-BPA water group, which indicated that the degradation of BPA via membranes effectively mitigates the effect on BPA on gastrointestinal tract.

## 1. Introduction

Humans are primarily exposed to bisphenol A (BPA) through oral contact due to its ubiquitous usage for food and drink packaging. The presence of BPA in various human samples, such as amniotic fluid, blood fetal tissues, and urine, is evidence of the significant BPA exposure through diet and drinking water. Chronic exposure to BPA in low doses alters some biological signaling, such as hormone–cell interaction, which has caused widespread concern for human health. Although the orally absorbed BPA comes into direct contact with the gut, most studies have focused on the effects of BPA on reproductive function and brain development. In the meantime, the effects of BPA on the small intestine (SI) are only reported by a few researchers [1,2,3], and its mechanism of action on the small intestine is still not well understood. In recent years, experimental evidence has shown that estrogens have been implicated in the establishment and regulation of the gut barrier [1]; however, the estrogenic signaling mechanisms in intestinal are clearly complex. Hence, xenoestrogens in BPA may exert their estrogenic activity to alter the intestinal function. 

Previously, Szymanska, Makowska, and Gonkowski [3] reported the impact of low and high doses of BPA (0.05 mg/kg and 0.5 mg/kg body weight/day, respectively) on the enteric nervous system (ENS) of the porcine ileum. The results observed changes that were probably connected with adaptive and protective mechanisms to maintain the homeostasis of the disturbed ileal by BPA even at low doses; this is suggestive of the impact of BPA on the ileum. In the meantime, Sarkar, Tarafder, and Paul [4] found significant inhibition on the movement of the duodenum ex vivo in a rat model by BPA with concentrations ranging from 10–320 μM. In addition, perinatal exposure to BPA has been associated with the development of gastrointestinal diseases in adult life, such as inflammatory bowel disease (IBD), colorectal cancer, and many more [5]. However, numerous factors influencing the effects BPA, especially the effect of low-level doses, on the digestive tract are still unknown. Interestingly, the investigation on the influence of perinatal exposure to BPA on gut physiology has shown BPA to decrease gut permeability at relatively low oral doses.

In the meantime, since the gastrointestinal tract (GIT) is directly in contact with ingested BPA, it is crucial to understand the effect of BPA exposure on the intestinal wall. The disruption of tight junction proteins (TJPs) may give an early sign for the detection of impaired function of the intestinal barrier. The disruption of the TJP integrity leads to an increase in epithelial permeability, which may trigger local or systemic inflammation and disease. Compromised intestinal barrier integrity is observed in both intestinal and systemic diseases, including inflammatory bowel diseases (IBDs), autoimmune diseases, and other metabolic diseases. The major types of tight junction proteins are the claudins and the occludins [6]. Occludin plays a role in the assembly and disassembly of TJPs [7], meanwhile claudins are the primary factor determinant of TJPs barrier function—they control the ion passage through the paracellular space [8]. Previous studies suggest that expression of claudin family members is altered in IBD [9]. The results demonstrated increased expressions of claudin-1 and -2 in IBD. Mees et al. [10] have suggested that claudin-1, -3, and -4 may be potential markers for diagnosing colorectal carcinoma. In human colorectal carcinoma, there is a significant increase of claudin-1, -3, and -4 expressions as compared with normal mucosa.

Since BPA has harmful effects on different organs of the body and one of the common sources of BPA is drinking water, there is a rising interest in developing a reliable water treatment system for removing BPA from water before it can be safely consumed. Interestingly, in our previous works [11,12], we successfully fabricated a visible light-driven nitrogen-doping titanium dioxide dual layer hollow fiber membrane (N-doped TiO_2_ DLHF) membrane for the degradation of BPA. Under certain circumstances, photocatalysis for the removal of contaminants from wastewater has demonstrated satisfactory results in degrading contaminants, as well as eliminating their harmful effects without the production of dangerous intermediates [13]. In the meantime, dual layer membrane configuration will provide simultaneous degradation and separation in a single unit. In the DLHF membrane configuration, the outer layer will provide a reaction site for the photocatalytic degradation in the presence of light irradiation, while the inner membrane layer will act as a separation layer. Interestingly, the incorporation of visible light active photocatalyst N-doped TiO_2_ on the membrane surface would allow the photocatalytic degradation activity, even under poor lighting sources. In this regard, the DLHF membrane had good removal of BPA, with 81.6% of degradation in the presence of visible light [14]. 

The hallmark for photocatalytic degradation of BPA in water is to remove the harmful effects of this chemical. Therefore, the current work is designed to study the effects of BPA-untreated and BPA-treated water on the important component of the SIB, SI tight junction proteins (TJPs) of both pregnant rats and their fetuses. A mother-to-infant transfer of maternal BPA in mice is consistent, with the potential of the chemical to cross the placental barrier and reach the fetus. Rats and humans have similar underlying physiology, organs, and body anatomy; hence, they often suffer from the same diseases. In this regard, the animal model study using rats would demonstrate a similar reaction to infection and injury, as both rats and human have nerve systems that act in the same way and use similar hormones in their bodies. The changes on the morphology of jejunum and ileum, as well as the expression of claudin proteins, will be observed to study whether reducing BPA levels through the photocatalytic degradation of N-doped TiO_2_ DLHF membrane can ameliorate the changes (if any). Gastrointestinal sections of tested pregnant rats in all groups, namely the jejunum and ileum, were gathered to assess the effects of BPA-untreated and BPA-treated water through histological analysis, and by measuring the expression of TJPs, and claudin-2, -3, and -4 protein. The differences in water intake and weight of each tested subject from were also observed to study the effect of BPA on the subjects’ general health status. In this regard, the in vivo model study will outline the post-treatment biological effects to describe the efficiency and rationale of the newly developed sustainable filter in areas where BPA levels in drinking water is high to reduce its side effect, thus helping people to have a better health.

## 2. Methodology

### 2.1. Materials for In-Vivo Models

BPA (CAS-number 80-05-7, purity ≥ 99%) as a model contaminant and Tween-80 used as a vehicle solution were purchased from Sigma Aldrich (Saint Louis MO, USA). Hematoxylin and eosin dyes (H&E) and xylene (Fisher Scientific, Leicestershire, UK), absolute ethanol (Merck, Branchburg, NJ, USA), formalin, and paraffin (Labchem, Zelienople, PA, USA) were purchased and used as instructed in standard protocols. 

### 2.2. Photocatalytic Removal of BPA by N-Doped TiO_2_ DLHF Membrane

Visible light-driven N-doped TiO_2_ photocatalytic DLHF membranes were fabricated using the co-extrusion method [12,15,16]. In our previous works [12,14], the photocatalytic degradation of BPA was carried out with DLHF membrane at N-doped TiO_2_/PVDF ratio was 0.5 due to its good sandwich-like structure and finger-like morphology at the inner and outer membrane region (Figure S1), good mechanical strength, and porosity of 13.3 MPa and 35.1%, respectively (Table S1). The characterization study also revealed that the membrane has good water permeability at 59.90 L/m^2^·h (Table S1) [14]. The experimental works to evaluate the degradation of BPA in contaminated water was performed using a batch-submerged membrane photoreactor, as described in our previous work [14]. For this, 30 hollow fibers were potted and fixed into a PVC adapter. The photocatalytic system was equipped with a visible light-emitting diode (LED 30W, Model: IP66) and an air diffuser to provide sufficient (O_2_) during the reaction. BPA at an initial concentration of 5 ± 0.12 ppm was used as a water pollutant model. In this case, a 5 ppm BPA initial feed was utilized based on the no-observed-adverse-effect level (NOAEL) for BPA, which was determined from a toxicology study in mice that looked at liver damage. The concentration was agreed by advisory bodies of the Food and Agriculture Organization of the United Nations (FAO) and World Health Organization (WHO) [17]. Prior to light irradiation, the membrane modules were immersed in the beaker, followed by the oxygenation for 2 h in the dark to achieve adsorption–desorption equilibrium. After that, the solution was exposed to visible light to promote photocatalytic degradation. Within the 6 h of the experiment, 10 mL of treated aliquots were collected at 30 min intervals (Ct). The materials were then analyzed using High Performance Liquid Chromatography (HPLC) with a UV detector (Agilent Technologies 1260 Affinity) to measure the concentration change of BPA throughout the experiment at 280 nm. The photocatalytic performance evaluations of the N-doped TiO_2_ DLHF reported that excellent removal of BPA was achieved after 360 min of visible light illumination (Figure S2) [14]. As a result, both the BPA-untreated water (feed) and the BPA-treated water (permeate) were stored in a BPA-free container for use in in vivo models. The concentration of BPA-untreated and BPA-treated water was 5.00 ± 0.12 ppm and 0.9 ± 0.07 ppm, respectively. 

### 2.3. Animal Care, BPA Exposure and Dissection Procedure

Animal care and BPA exposure was conducted as described in our previous work (UiTM CARE: 254/2018(3 August 2018)), and a proof/certificate of approval is available upon request (Kamaludin et al., 2020). In total, 15 male and 30 female Sprague–Dawley rats aged between 150–180 days were purchased. Then, two female rats were caged with one male rat for the mating process. Following the vaginal smear test after the mating process, the pregnant rats were randomly divided into three groups, namely vehicle control Tween 80 (VHC TN80), BPA-untreated (UT), and BPA-treated (T). Pregnant rats were housed individually and administered with a designated treatment (vehicle control Tween 80 water, BPA-untreated water, and BPA-treated water, as previously described, by oral gavage beginning on day 2 of pregnancy until 21 day of pregnancy. Weight and water (treatment) intake were likewise monitored for each rat from all groups for 21 days, and the observation was recorded on treatment days (TD) of 2, 7, 14, and 21.

#### 2.3.1. Hematoxylin & Eosin (H & E) Staining

The dissection procedures were as described in our previous works [14]. The jejunum and ileum samples from each group were processed for 24 h in a tissue processing machine, then implanted in metal molds filled with paraffin wax, and sectioned at a thickness of 4 µm. Paraffin sections were prepared according to the protocol provided (Fisher Scientific, Leicestershire, UK),). The resultant sections were mounted in DPX mountant, and were covered with glass coverslips.

#### 2.3.2. Western Blot Analysis

Briefly, protein extraction was performed using a lysis cocktail, including a radioimmunoprecipitation assay (RIPA) buffer (Thermo Fisher Scientific, Waltham, MA, USA), 0.5M EDTA (Thermo Fisher Scientific, Waltham, MA, USA) and a protease inhibitor (Thermo Fisher Scientific, Waltham, MA, USA) cocktail. The lysed tissues were then homogenized. Jejunum and ileum samples were vortexed, followed by centrifugation at 13,000× *g*. Then, 15 µg of the protein samples was electrophoresed in 10% SDS-PAGE, and then electro transferred onto nitrocellulose membranes (Bio-Rad). The membranes were then blocked in Superblock blocking buffer in TBS (Thermo Fisher Scientific, Waltham, MA, USA). The membranes were incubated with anti-claudin-2, -3, and -4 (Thermo Fisher Scientific, Massachusetts, USA) at 4 °C, followed by washing with PBS/Tween prior to the incubation with a secondary antibody (Thermo Fisher Scientific, Waltham, MA, USA) at room temperature. Antibody binding was detected using a Supersignal West Pico chemiluminescence substrate (Thermo Fisher Scientific, Waltham, MA, USA).

### 2.4. Statistical Analysis

Data were analyzed using SPSS software package (IBM Corp. Released 2017. IBM SPSS Statistics for Windows, Version 25.0. Armonk, NY: IBM Corp.) where ANOVA was used for parametric data and Kruskall–Wallis for non-parametric data. The statistically significance was set at *p* < 0.05, *n* = 3.

## 3. Results and Discussion

### 3.1. General Health of Rat Fetus and Mother

**Figure 1:**
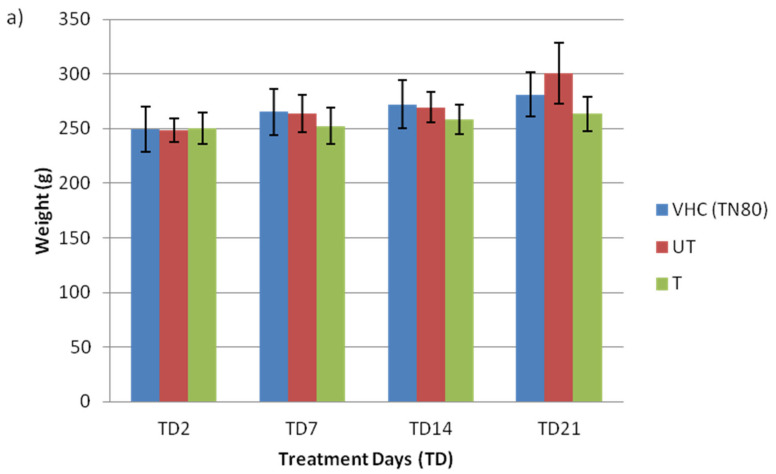

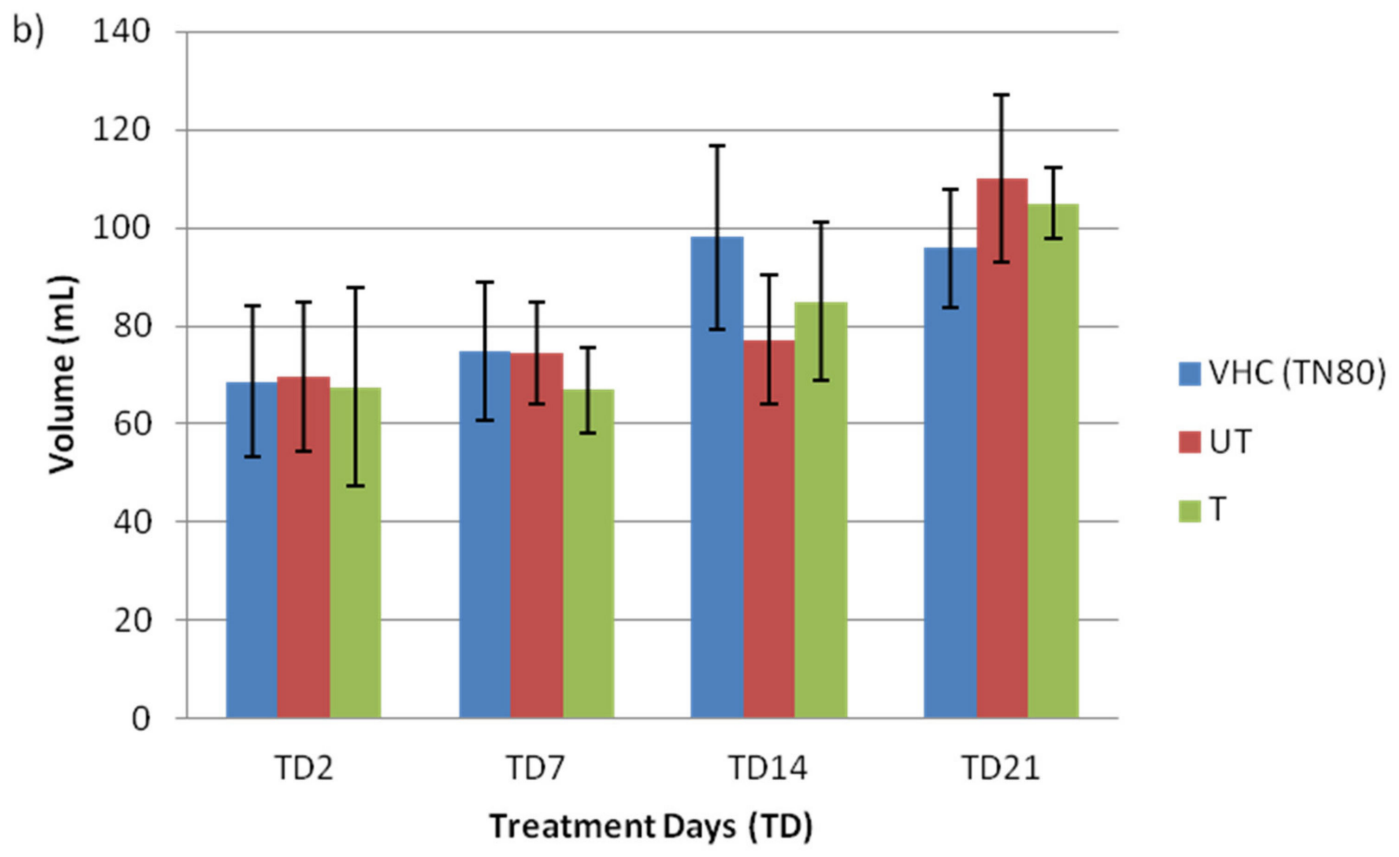
Effects of BPA on (**a**) body weight and (**b**) drinking patterns of pregnant rats (*n* = 3–5 rats per group).

The BPA-untreated (initial concentration of 5.00 ± 0.12 ppm) and BPA-treated water (concentration after photodegradation of 0.9 ± 0.07 ppm) from Section 3.2 was used as a treatment in animal models to be administered via drinking water. Photocatalytic activity allows for high removal of BPA; however, many by-products are released despite their satisfactory removal results. The intermediate compound formed during the degradation process could have an estrogenic activity higher than their mother compound. Therefore, the potential of this treatment in completely removing BPA and its by-product should also be given attention to help to rationalize the use of the newly developed membrane technologies in reducing the level of BPA, as well as their estrogenicity. Rats were administered with vehicle control Tween 80 (VHC TN80), BPA-untreated (UT), or BPA-treated (T) water by N-doped TiO_2_ DLHF membrane for 21 days. Figure 1 depicts the effects of BPA on weight and water intake of treated rats from all groups. There is no significant weight gain (Figure 1a), nor were drinking patterns of pregnant rats (Figure 1b) observed in tested rats from all groups during the BPA treatment days (TD). Interestingly, the amount of water intake increased up to 60–100 mL/day at the end of the pregnancy. Similarly, comparable average water consumption in BPA (47.4 ± 3.0 mL/day) and control groups of (46.4 ± 3.7 mL/day) was reported previously [18]. Rats treated with BPA had identical behavior, hunger, and body weight increases as the control group. This discovery is in line with a prior study that found similar results, where experimental animals did not demonstrate any clinical indications of intoxication following BPA treatment [3]. 

Table 1 shows the number of fetuses produced from VHC and BPA-exposed pregnant mothers and their gender distribution (*n* = 3–5 rats per group). Generally, the lowest number of pups produced was 5, and the highest number of pups was 10. From Table 1, it also can be seen that the number of female pups was higher in all groups. However, the difference in the number and gender distribution of pups was not significant due to the inconsistent number of pregnant mothers from each group. This inconsistency was due to external factors, such as the pregnant mothers dying during the experimental observation, early delivery, or miscarriage. Previous study [19] found that prenatal BPA exposure had no effect on the sex ratio of newborn pups, where the proportion of males per litter was identical in control (53%) in comparison to the BPA-exposed animals (50%). Interestingly, BPA has been shown to affect the degree of masculinization or feminization of external genitalia; time of vaginal opening (indicating the start of female puberty); and the onset of the estrous cycle (indicating sexual maturity) [20]. At low doses, perinatal BPA exposure has been shown to negatively impact on both male and female rat sexual development. Male and female anogenital distance (AGD) at birth was significantly reduced at dosages of 250 g/kg and 25 g/kg bw per day and above, respectively. This finding suggests endocrine impacts of BPA on sexual development [21]. In addition, ovary, uterine, and vaginal weights (showing estrogenic activity); fertilization rate; egg shape; and number of live-born neonates per litter have been demonstrated to be affected by BPA in experimental experiments [22]. 

**Figure 2:**
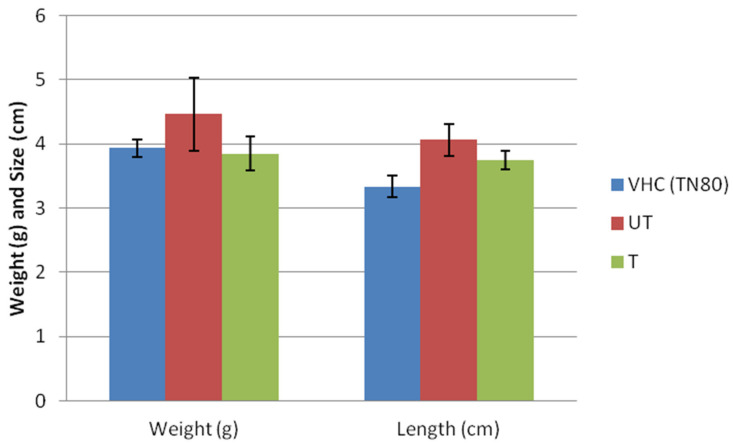
Effects of BPA on weight and length of fetus from vehicle (VHC TN80), BPA-untreated (UT), and BPA-treated (T) groups.

The changes in fetuses’ weight and size can be observed in Figure 2. There are increases in the weight of fetuses from untreated-BPA group (4.5 g ± 0.57), compared to the VHC (3.9 g ± 0.13) and treated-BPA groups (3.9 g ± 0.26). Similar patterns were observed in the length of fetuses, in which length increase was observed in the untreated-BPA group (4.1 cm ± 0.25), compared to the VHC (3.3 cm ± 0.17) and treated-BPA groups (3.8 cm ± 0.14). However, the differences in either both parameters were not significant between fetuses from the control, untreated-BPA, and treated-BPA groups. Interestingly, Reddivari et al. [23] reported that perinatal BPA exposure did not significantly influence litter size, survival, birth weight, or sex ratio. 

### 3.2. Changes in Morphology of Jejunum and Ileum and Claudin Protein Expression

**Figure 3:**
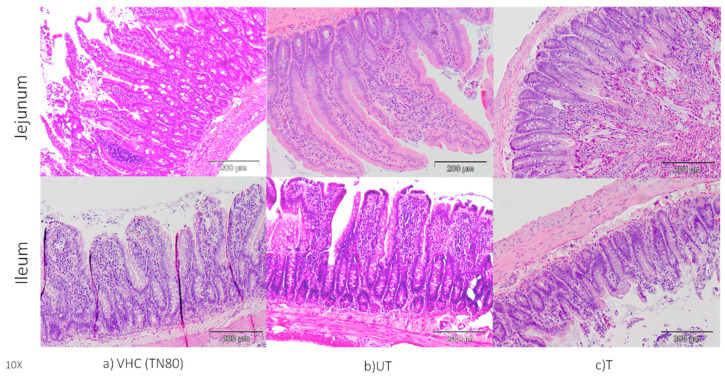
Histopathological staining of intestinal collected from rats (**a**) VHC group (**b**) BPA-untreated (UT) group, and (**c**) BPA-treated (T) group (*n* = 3–5) (10× magnification).

As the first organ in contact with food and drinks, the small intestines serves as a first line of defense in preventing hazardous chemicals from entering the lumen. Due to BPA xenoestrogen activity, it may have effects on the intestinal barrier. The effect of BPA on morphology of the jejunum and ileum of pregnant rats are shown via H&E, as represented in Figure 3. As can be seen from the figure, BPA altered the normal morphology of the jejunum and ileum. In the BPA-untreated group, typical villi projections were disturbed and lost their morphology, whereas the VHC TN80 group had normal villi projections. Interestingly, the villi projection in the BPA-treated group was identical to that in the VHC group. This finding clearly showed that BPA-treated water by photocatalytic N-doped TiO_2_ DLHF membrane successfully removes the detrimental effects of BPA. Similarly, Apaydin et al. [24] found various pathological abnormalities in the small intestine tissues of BPA-treated rats, including necrosis and degenerative changes. In the meantime, BPA at 1/25 of oral LD_50_ showed that BPA induces edema and necrosis in the small intestine of tested rats due to oxidative damage [25]. Another study [3] was performed to investigate the effects of low dose (LD) and high dose (HD) BPA on the ileum of tested porcine, which revealed that the morphology of the intestinal tract was still intact, with normal villi morphology, as well as the presence of eosinophilic granulocytes and plasmatic cells in the mucosal and submucosal lining. However, an increased number of eosinophils with visible inflammatory cells were observed in the intestinal crypts in the high dose group, compared to the low dose and control groups. 

**Figure 4:**
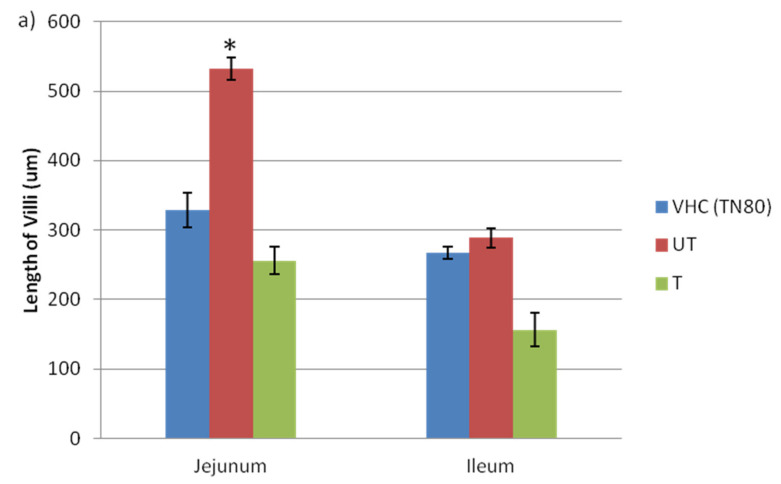

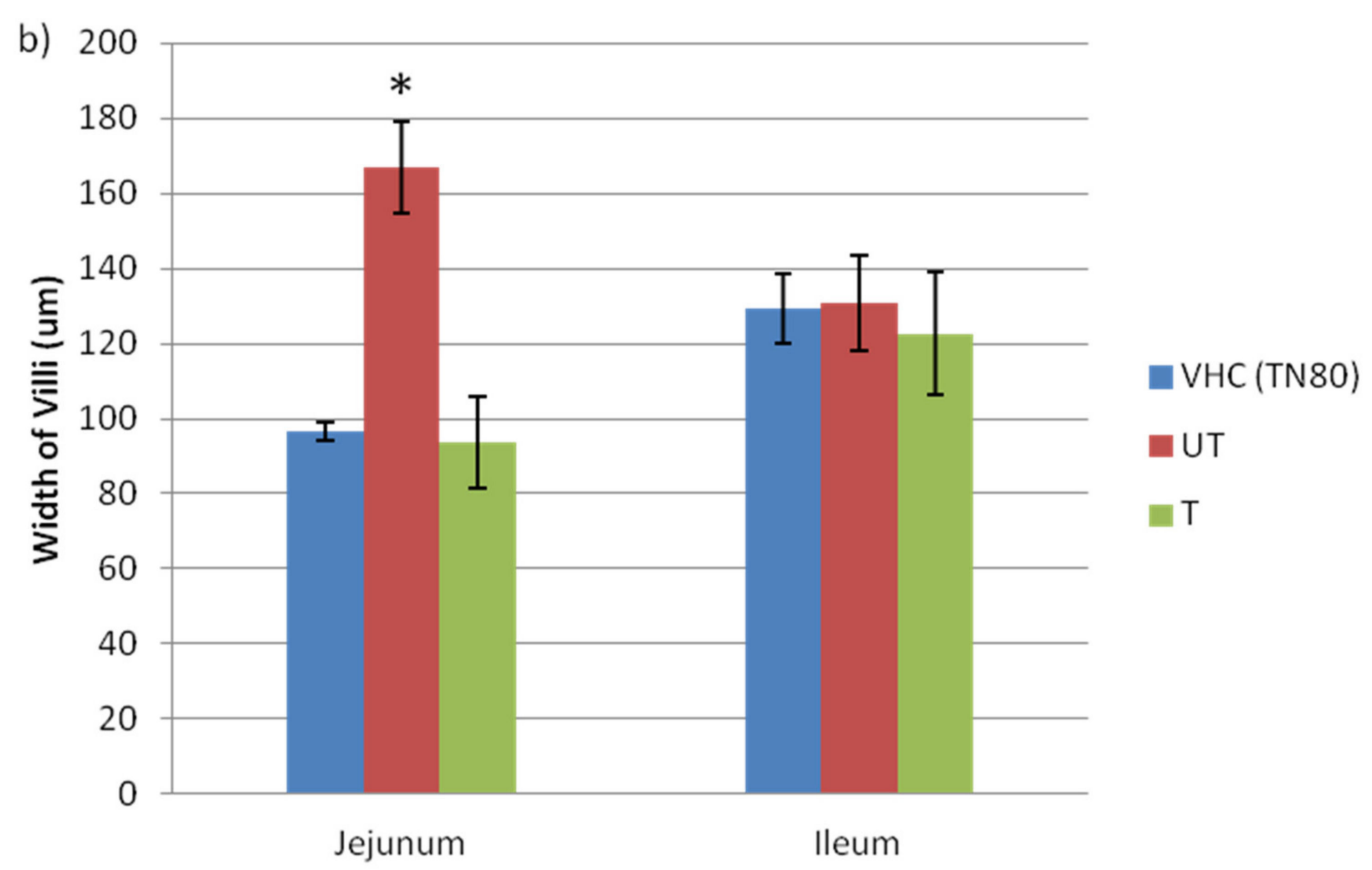
Effects of BPA on general parameters of villi in the jejunum and ileum of pregnant rats (**a**). Length of villi (**b**). Width of villi. (*n* = 3–5 rats per group). * Significant as compared to VHC group at *p* < 0.05.

Anything that causes inflammation of the villi in the small intestine can affect digestion and absorption, hence the villi of the jejunum and ileum were further analyzed. The length, width, crypts, and the number of goblet cells were observed and recorded. The length and width of villi in the jejunum and ileum of VHC TN80, BPA-untreated, and BPA-treated exposed pregnant rats are shown in Figure 4. Significant increases in the length (Figure 4a) and width (Figure 4b) of villi in the jejunum of the BPA-untreated group (532.5 ± 16.23, 166.75 ± 22.11, respectively) compared to the VHC TN80 (328.8 ± 25.09, 96.70 ± 2.42, respectively) and BPA-treated group (256.3 ± 19.5, 93.47 ± 12.21, respectively) of the ileum were observed. In contrast, there were no significant differences in the length and width of villi in the ileum in all groups: BPA-untreated, BPA-treated (156.14 ± 24.08, 122.69 ± 16.28, respectively), and VHC TN80 group. 

**Figure 5:**
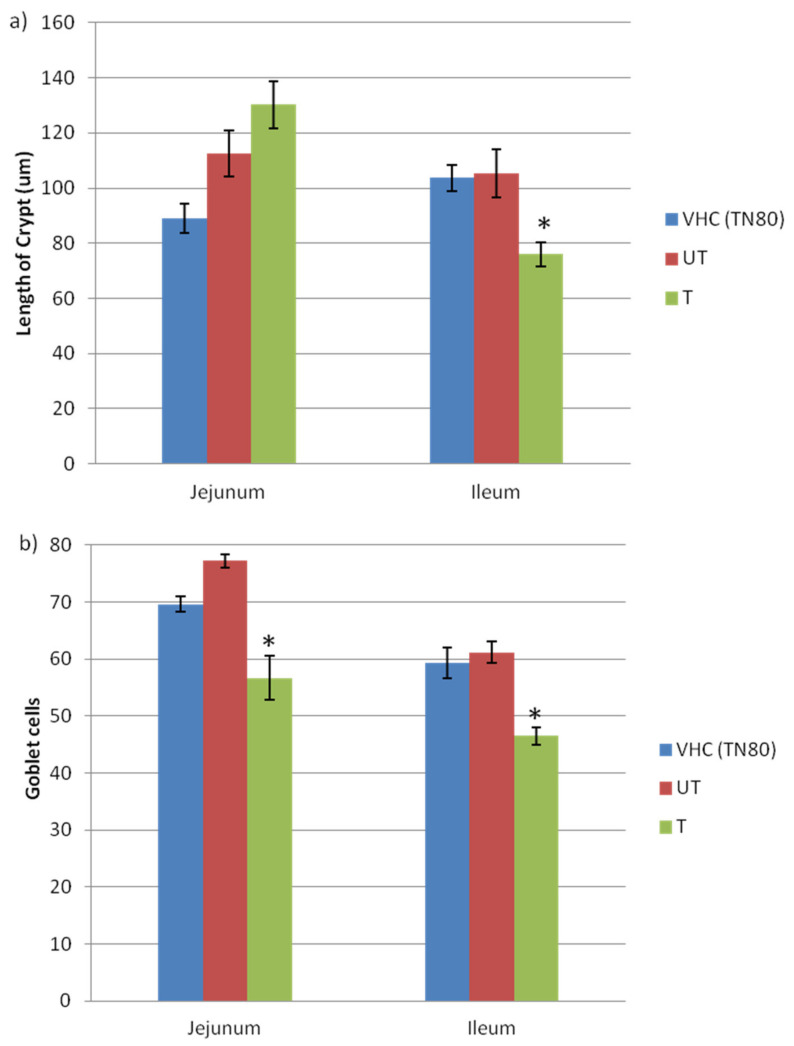
Effects of BPA on (**a**) crypt and (**b**) goblet cells of villi in the jejunum and ileum of pregnant rats (*n* = 3–5 rats per group). * Significant as compared to VHC group at *p* < 0.05.

Crypts (of Lieberkuhn) are moat-like invaginations of the epithelium surrounding the villi, which are mostly lined by younger epithelial cells that are principally involved in secretion [26]. Many essential cells, including those involved in host defence and signalling, are found in the crypts. In addition, stem cells are found around the base of the crypts, where they divide constantly, and represent the source of all epithelial cells in the crypts and on the villi [27]. Further investigation on the length of crypt in the jejunum and ileum of pregnant rats are shown in Figure 5a. In the jejunum, there is an increase in the length of crypts in the BPA-untreated (130.28 ± 8.63) and BPA-treated (112.53 ± 8.38) pregnant mothers, as compared to the VHC TN80 group (88.92 ± 5.33). In contrast, there are no significant differences in the crypts of the ileum between VHC (103.68 ± 4.84) and BPA-untreated exposed (105.23 ± 8.72) pregnant mothers; however, the crypts of the ileum in BPA-treated exposed (76.04 ± 4.38) pregnant mothers are significantly decreased, as compared to the untreated BPA-exposed pregnant mothers. Many important cells reside in the crypts, including those involved in host defense and signaling, hence changes in the crypt might indicate the inflammation occurred in the jejunum and ileum.

In the meantime, Figure 5b demonstrates the changes in the number of goblet cell in the jejunum and ileum of pregnant rats from all groups. It can be seen that the number of Goblet cells were high in the BPA-untreated group of the jejunum (79.83 ± 2.11) and ileum (61.19 ± 1.87) of the BPA-untreated group of exposed pregnant rats. In contrast, the number of goblet cells was significantly lower in the BPA-treated group of both the jejunum (56.71 ± 3.93) and ileum (46.50 ± 1.56). As goblet cells produce acid glycoproteins of the mucin type that are hydrated and cross-linked to form mucus, their main function is to protect and lubricate the lining of intestine. Therefore, these findings show that BPA exposure is harmful to the small intestine, which consequently further affects human health.

H&E staining results in this present study demonstrated that exposure to BPA altered the epithelial barrier in the jejunum and ileum of pregnant mothers. Similarly, Szymanska, Makowska and Gonkowski [3] reported an increased number of eosinophils in the HD of a BPA sample, which indicated that there were inflammations in the intestinal sections of the treated sample. In the meantime, Liu et al. [28] showed that maternal BPA exposure significantly decreased the ratio of villus height to crypt depth in the jejunum of newborn and weaning pigs. This finding may indicate less proliferation and differentiation of intestinal epithelial cells, thereby reflecting the suppressing capacity of the small intestine for nutrient digestion and absorption [29]. To date, limited studies have reported the chronic effect of BPA on the jejunum and ileum of small intestine. However, Dixit et al. [30] observed that chronic exposure of BPA affects the intestinal smooth muscle contractile mechanisms. The effect of BPA on the frequency of contraction was not uniform, which depicts that the small and large intestine may be differentially affected by BPA. In this regard, the frequency was significantly decreased in colon, while the ileum remained unaffected. Therefore, the large intestine appears to be more vulnerable to BPA-induced adverse effects, in comparison to the small intestine.

### 3.3. The Expression of Claudin Protein in Jejunum and Ileum

**Figure 6:**
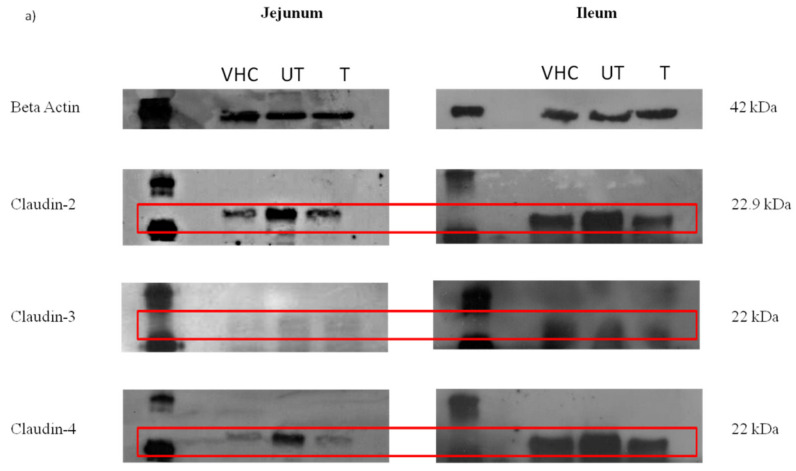

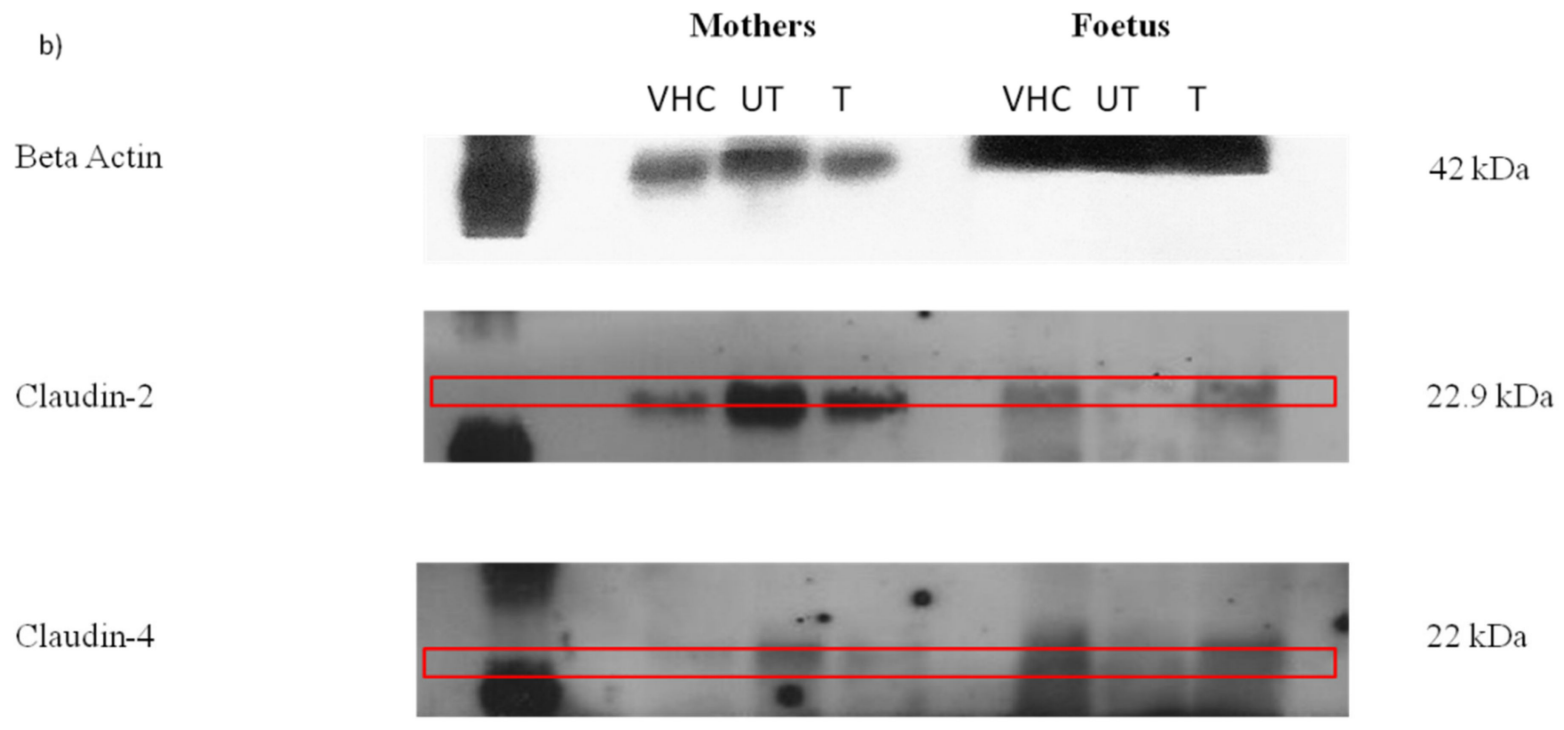
Representative diagram of Western blotting of the jejunum and ileum collected from (**a**) pregnant rats and (**b**) fetuses from the VHC TN80, BPA-untreated (UT) water, and BPA-treated (T) water groups (*n* = 3–5 rats per group).

The expressions of claudin-2 and -4 in the ileum and jejunum were qualitatively analyzed by Western blotting, as shown in the Figure 6a. The claudin-2 and -4 protein expression was highest in the ileum, with relatively high expression in the BPA-untreated group, as compared with the VHC TN80 and BPA-treated group. In the meantime, the protein expression of claudin-3 was relatively low in the ileum of all groups, and was nearly undetectable in the jejunum of all groups. The finding in this present study is in agreement with previous investigations which reported that the expression of the claudin-2 protein was relatively high in the colon, duodenum, and ileum, compared to the other organ [31,32]. Another study reported a significant decrease in claudin-3 expression in the uterine luminal epithelial cells of both low (BPA-L) and high dosage (BPA-H) groups and a significant increase in claudin-4 in BPA-L, as compared to the control group [33]. 

As for fetuses, the expression of claudin in the intestines of fetuses from all groups is shown in the Figure 6b. As can be seen from the figure, in the BPA-treated group, the expression of claudin-2 and -4 in the intestines of fetuses was similar with the expression in the other two groups, whereas the expression of claudin-2 in the BPA-untreated group was relatively lower. In contrast, the expression of claudin-3 was undetectable in the intestines of fetuses from all groups. From the results, the claudin-2 and claudin-4 expressions in the intestines of fetuses were contrary from the mothers. This might be due to the difference in the intestinal structure of both fetus and mother. 

Claudins were commonly found in both small and large intestine erythrocytes. Changes in claudin proteins influence epithelial and mucosal homeostasis by altering epithelial barrier activities. Therefore, disruption of the tight junction barrier function and changes in permeability properties has been shown to be associated with various pathologic conditions [34]. These findings are consistent with prior reports of claudin-2 overexpression in the intestinal epithelia, which is associated with increased colonic crypt length and total intestine elongation [35]. Another report on the increased expression of claudin-2 in the intestinal tract has been associated with ulcerative colitis [36] and Crohn’s disease [37]. In addition, claudin-3 expression has also been found to be higher in the mucosal tissue of patients with inflammatory bowel disease. [38]. Furthermore, significantly high expression of claudin-3 and -4 in endometrial carcinogenesis strongly recommended their potential value as diagnostic and prognostic biomarkers in normal, hyperplastic, and malignant endometrial tissue [39]. In the meantime, claudin-4 is overexpressed in pancreatic cancer, and associated with decreased invasiveness in vitro and in vivo [40]. 

## 4. Conclusions

The integrity of the intestinal epithelial is crucial for nutrition absorption and the body’s defense against infections. It is even more important during pregnancy, as developing fetuses are mainly dependent on nutritional support from their mother. Here, BPA causes changes in the structure of the jejunum and ileum, especially the finger-like villi projections. In the BPA-treated group, normal villi projections were preserved and intact. Furthermore, a lower number of goblet cells lower in both the jejunum and ileum in treated BPA-exposed pregnant rats indicated that intestinal epithelial cells experience less inflammation, as compared to the BPA-untreated group. Furthermore, BPA exposure may alter the expression of claudin-2, claudin-3 and claudin-4 proteins in the ileum and jejunum, with a high expression of investigated claudins in BPA-untreated group. In contrast, the BPA-treated group was observed, with low expressions of claudin-2, claudin-3, and claudin-4 protein expressions in both the jejunum and ileum of pregnant mothers. Most importantly, the results of the BPA-treated group were similar to those of the VHC group, indicating that the toxicity effect of the BPA on the small intestine has been reduced after the treatment process. 

## Figures and Tables

**Table 1:** Number of fetuses and gender distribution.

Group	No. Pregnant Rats	No. of Fetuses					Total No. of Foetus	Gender Distribution	
		Mum 1	Mum 2	Mum 3	Mum 4	Mum 5		Male	Female
VHC (TN80)	3	8	10	6	Nil	Nil	24	11	13
UT	5	8	5	7	7	5	32	14	18
T	4	10	10	8	10	Nil	38	14	24

## Data Availability

The raw/processed data required to reproduce these findings cannot be shared at this time, as the data also form part of an ongoing study.

## Supplementary Materials

The following supporting information can be downloaded at: https://www.mdpi.com/article/10.3390/toxics10060296/s1, Figure S1: SEM cross-section morphological analysis of DLHF membranes; (a) N-doped TiO2 DLHF and (b) TiO2-P25 DLHF (Kamaludin et al., 2020); Figure S2: Photocatalytic degradation of BPA by N-doped TiO2 and TiO2-P25 DLHF membrane under visible light irradiation (Kamaludin et al., 2020); Table S1: Performance of N-doped TiO2 DLHF in comparison with TiO2-P25 DLHF (Kamaludin et al., 2020).
