# Supplementary material for: The Effect of BPA-Treated Water on the Small Intestine via an In Vivo Study
Source: Toxics. 2022 May 30;10(6):296. doi: 10.3390/toxics10060296 (PMC9228272; doi:10.3390/toxics10060296)
Supplement: Supplementary file 1 [file toxics-10-00296-s001.zip › toxics-1720321-supplementary.pdf]

toxics

Supplementary Materials: The Effect of BPA-Treated Water on the Small Intestine via an In Vivo Study

Roziana Kamaludin, Zatilfarihiah Rasdi, Mohd Hafiz Dzarfan Othman, Siti Hamimah Sheikh Abdul Kadir, Mohd Yusri Idorus, Jesmine Khan, Wan Nor I'zzah Wan Mohamad Zain, Ahmad Fauzi Ismail, Mukhlis A. Rahman and Juhana Jaafar

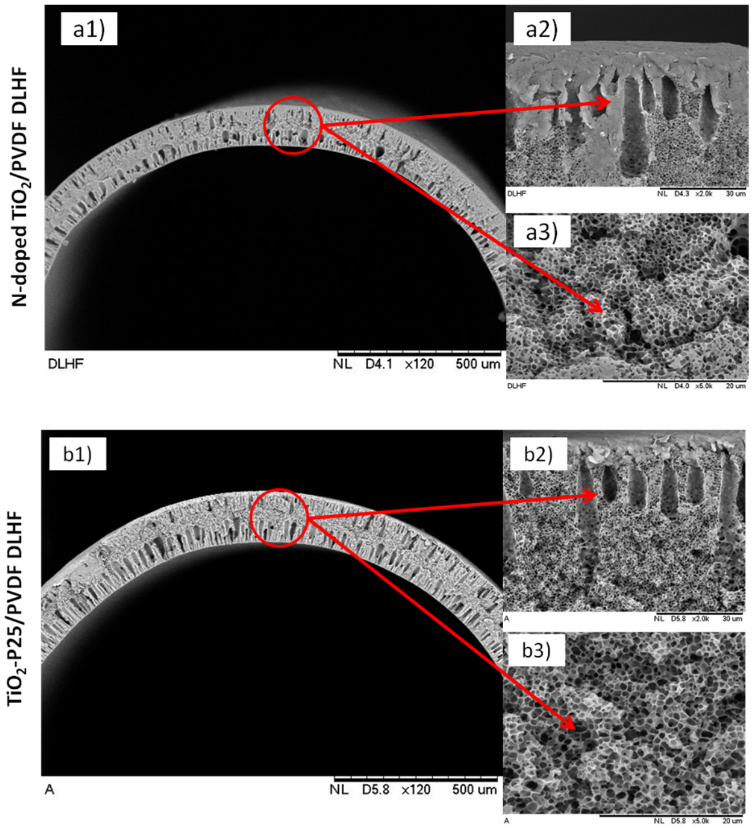

Figure S1. SEM cross-section morphological analysis of DLHF membranes; a) N-doped TiO_2 DLHF and b) TiO_2-P25 DLHF (Kamaludin et al., 2020).

Table S1. Performance of N-doped TiO_2 DLHF in comparison with TiO_2-P25 DLHF (Kamaludin et al., 2020).

Configurations	N-doped TiO_2/PVDF DLHF	TiO_2-P25/PVDF DLHF
**Tensile Strength (MPa) :**	13.3 ± 0.24	14.5 ± 1.54
**Porosity:**	35.1	37.9
**Water Flux (L/m²·h):**	59.90	67.19

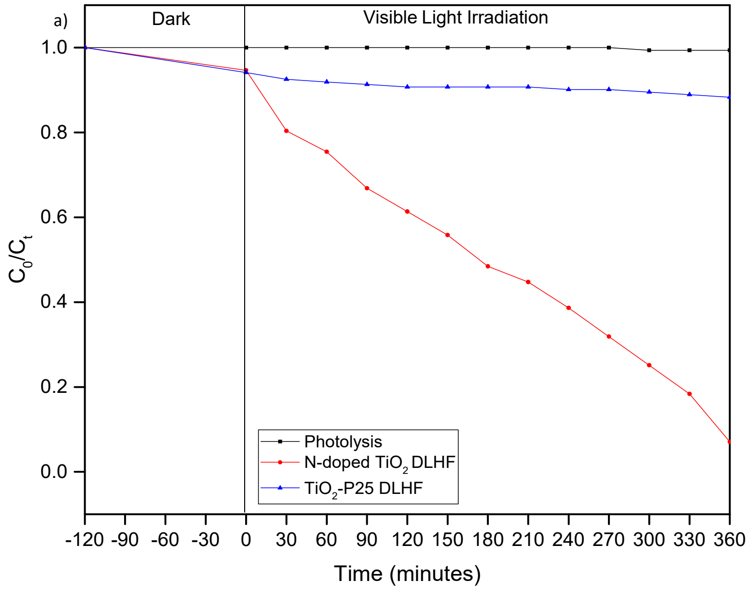

Figure S2. Photocatalytic degradation of BPA by N-doped TiO_2 and TiO_2-P25 DLHF membrane under visible light irradiation (Kamaludin et al., 2020).
